# Risk of venous occlusion after lead laser extraction preventing future lead implantation

**DOI:** 10.1186/s13019-021-01706-5

**Published:** 2021-10-30

**Authors:** Sameer Al-Maisary, Jamila Kremer, Gabrielle Romano, Matthias Karck, Raffaele De Simone

**Affiliations:** Department of Cardiac Surgery, Heidelberg University Hospital, INF 420, 69120 Heidelberg, Germany

**Keywords:** Lead laser extraction, Venous occlusion, Cardiac implantable electronic devices, Thrombosis, Anticoagulation

## Abstract

**Background:**

Lead laser extraction is a well-established method for removing unwanted leads with low morbidity and mortality.

**Objective:**

In this observational study, we documented our experience with venous occlusion after lead laser extraction.

**Methods:**

Retrospective data of patients who underwent lead laser extraction between May 2010 and August 2018 was analyzed. Two subgroups of patients were identified. First group represented patients after lead laser extraction who suffered postoperative venous occlusion. Second group represents patients after lead laser extraction, who has documented patent venous access after lead laser extraction.

**Results:**

219 patients underwent percutaneous laser lead extraction. The mean age of patients was 65 ± 14 years. Of these patients, 74% were male. The Most common indication for extraction was Nonfunctional lead (45.2%, n = 99) followed by pocket infection with 33.8% and endocarditis (17.3%). A total number of 447 leads underwent laser extraction. In 7.8% of the patients, lead extraction was partially successful and lead extraction was not successful in only 2.3% of the patients. Only 13 patients developed a documented venous occlusion postoperatively and 26 patients has documented absence of venous occlusion, of whom 17 were under oral anticoagulation.

**Conclusion:**

Lead laser extraction may lead to venous occlusion, which is mostly asymptomatic but it prevents future lead implantation. The use of oral anticoagulant may prevent postoperative venous occlusion.

## Background

The implantation of Cardiac implantable electronic devices (CIED) has increased in the last decades due the benefit of increasing survival and improving the quality of life in patients with cardiac rhythm disorders. This wide spread use is also associated with an increase in post-implantation complication of which is infection and dysfunction the most prominent ones due to its effect on therapy strategies and the life expectancy of the affected patients due to the significant increase in morbidity and mortality [1, 2]. The infection rate of CEIDs ranges between 1 and 7%. On the other hand, the rate of dysfunctional leads reaches 7% [3–7]. Device removal is the gold standard for treating CIED infection because conservative therapies usually fail [8, 9]. Extraction of infected or dysfunctional leads was performed using different strategies, starting with simple traction or continuous traction to using polymer or metal sheath systems with or without locking stylets. More recently, new devices were introduced using laser or radiofrequency. These are cost and time-consuming procedures needing highly trained and experienced operators. The percutaneous lead extraction is also accompanied with serious life-threatening complications. The evidence of venous injury during lead laser extraction was documented and until now and no serious consequences were presented [10]. We report our experience in CEID extraction using the 80 Hz high frequency laser sheaths to evaluate its safety and efficacy and the risk of venous thrombosis following extraction.

## Materials and methods

This is a retrospective observational study of consecutive patients who underwent laser-assisted lead extraction (using GlideLight laser sheath, Spectranetics Corporation, Colorado Springs, USA) between May 2010 and August 2018. The indications for lead extraction varied between pocket infection, device-related endocarditis, pain, and abandoned leads. Patients were referred from external hospitals or from our electrophysiological outpatient clinic. Indications for lead extraction were Pocket infection, Device-related endocarditis, Pain, abandoned or non-functioning leads and blood vessel obstruction. Pocket infection was defined as redness with or without purulent discharge from the device pocket or device erosion which may be accompanied by pain. Device-related endocarditis was defined as persistent bacteremia or sepsis in the absence of another identifiable source or the presence of vegetations on the leads or the valves. Pain related to CEID or Leads was considered also as an indication for extraction. Extraction of abandoned or non-functioning leads is performed if they produce obstruction to a blood vessel or if a new lead implantation will increase the burden of the total lead number. The use of Laser Sheath was indicated if the removal of the leads under simple traction is not successful. All procedures were performed under general anesthesia with continuous arterial blood pressure monitoring. Under fluoroscopic guidance, the lead extraction starts by inserting a lead locking stylet into the inner coil lumen then a suture is tied around the insulation and the locking stylet. After that, the laser sheath (Glide Light 80 Hz, 14 or 16 French) was advanced over the lead until the locking stylet emerges from the other side of the laser sheath. Laser is then applied while gradually advancing the sheath over the lead under traction until the lead is freed. If all leads are removed, the procedure was considered successful. Transesophageal echocardiography was used to monitor the procedure. If the has no infection, device reimplantation was performed from the same side after advancing a guide wire through laser sheath. In patients with local or systemic infection, no device would be implanted unless the patient is pace-maker dependent. In this case an epicardial pace maker lead is implanted though a an inferior pericardiotomy as described by Al-Maisary et al. [11] and after remission of infection, the patient would be re-evaluated for dual-chamber pace-maker implantation. An electronic database of all patients was retrospectively analyzed to obtain the needed information. The collected data includes patients´ characteristics, indications for lead extraction, type of lead and age of lead, details of procedure, intra- and postoperative complications, in-Hospital mortality up to 30 days and pre- and postoperative medications. Removal of all leads was considered as procedural success. Removal of some leads was considered as partial success. Procedural failure was defined as the inability to remove any lead. Two subgroups were identified for investigating venous occlusion. The first group represents the venous occlusion group. Venous occlusion was diagnosed if a patient shows clinical symptoms of venous thrombosis of arm veins confirmed whether using duplex ultrasound, Phlebography or Computer tomography angiography. Also, patients who underwent phlebography or duplex ultrasound examination, after laser lead extraction, for preoperative evaluation prior to the implantation of pacemaker leads with newly developed venous occlusion, were included. The second group (control group) included each patient who underwent phlebography, duplex ultrasound showing patent vessels after laser extraction or who successfully received new leads or indwelling catheters in the same side of laser extraction and differences between these two groups were investigated. Data analysis for Demographic Data, medical history, and lead-related characteristics were presented as frequency (%) for categorical variables and as mean plus or minus standard deviation for normally distributed continuous variables. The study protocol was approved by the institutional research ethics board at Heidelberg University.

## Results

From May 1, 2010 to Mars 25, 2018 a total of 219 patients underwent percutaneous laser lead extraction in our center. Patient characteristics can be seen in Table 1.

**Table 1:** Demographics

	Total	Venous occlusion group	Control Group
Patients	n = 219	13	26
Age	65 ± 14	57 ± 17	61 ± 18
Sex, % male (n = 163)	74.4	4.5 (10)	6.8 (15)
Body mass index	27.6 ± 10.5	25.0 ± 5.6	26.8 ± 4.4
Diabetes mellitus, % (n)	31.9% (70)	1.36 (3)	3.65 (8)
EF, %	42.05 ± 14.5	40.8 ± 16.0	34.9 ± 10.1
NYHA functional class III	62 (28.3%)	1.5 ± 0.8	2.1 ± 0.9
Coronary artery disease	106 (48.4%)	4 (1.8%)	14 (6.4%)
Arterial hypertension	187 (85.4%)	11 (5.0%)	24 (10.9%)
Renal insufficiency	28 (12.8%)	0	5 (2.2%)
ICD	162 (73.97%)	10 (4.5%)	22 (10.0%)

ICD, implantable cardioverter-defibrillator

The mean age of patients was 65 ± 14 years. Of these patients, 163 were male (74.4%). The body mass index was 27.6 ± 10.5 and 31.9% had diabetes mellitus. The ejection fraction was 42.05 ± 14.5% and only 10 (4.6%) patients has NYHA class IV. Coronary heart disease was seen in 106 (48.4%) of patients and 85.4% of patient has arterial hypertension. Only 12.8% presented with renal insufficiency. Most of the patients (73.97%) had an ICD. The indications for lead extraction in this study are presented in Table 2.

**Table 2:** Indications for lead extraction

	Total	Venous occlusion group	Control group
Pocket infection	74 (33.8%)	2 (0.9%)	6 (2.7%)
Endocarditis	38 (17.3%)	1 (0.4%)	4 (1.8%)
Bacteremia or Sepsis	25 (11.4%)	1 (0.4%)	3 (1.3%)
Functional abandoned lead	9 (4.1%)	0	2 (0.9%)
Nonfunctional lead	99 (45.2%)	9 (4.1%)	17 (7.7%)
Pain at device or insertion site	2 (0.91%)	1 (0.4%)	0
Venous stasis or occlusion	4 (1.82%)	0	0

The Most common indication for extraction was Nonfunctional lead (45.2%, n = 99) followed by pocket infection with 33.8% and endocarditis (17.3%). Sepsis or bacteremia related lead or device implantation was present in 11.4% of patients. Functional abandoned leads were an indication for lead extraction in 4.1% of patients. Only 1.82% of patients had venous stasis or occlusion and 2 patients asked for device remove due to chronic pain. A total number of 447 leads were underwent laser extraction with a mean time from implantation of 100.40 ± 64.23 months. Single coil ventricular leads were the most frequently encountered leads (28.63%) followed by atrial leads and dual coil leads. Coronary Sinus leads were the least frequently removed leads (6.7%). Most of the Leads (91.5%) were extracted successfully. For patients, the percentage of successful removal of all leads was 90%, for partial removal 7.8% and only in 2.3% of the patients had no leads removed with no statistical significance. Lead Demographics are shown in Table 3.

**Table 3:** Lead demographics

	Total	Venous occlusion group	Control group
Number of treated leads	447	30	50
Mean time from implantation, months	100.40 ± 64.23	87.7 ± 43.9	75.2 ± 48.2
*Lead type, n (% within the group)*			
Atria lead	122 (27.29%)	8 (26.6%)	11 (22.0%)
Ventricular pacemaker lead	101 (22.59%)	6 (20%9	8 (16.0%)
Coronary sinus lead	30 (6.71%)	4 (13.3%)	5 (10.0%)
Single coil lead	128 (28.63%)	7 (23.3%)	20 (40.0%)
Dual coil lead	66 (14.76%)	5 (16.7%)	6 (12.0%)

Only one patient suffered from superior vena cava tier requiring open surgery. One patient died due to pulmonary embolism. Thirteen patients have developed a documented venous occlusion postoperatively and 26 patients has documented absence of venous occlusion. A total of 30 leads was extracted (2.3 lead per patient) in the venous occlusion group and 50 (1.9 lead per patient) in the control group. Most of the extracted leads were single coil ventricular leads (28.6%) followed by atrial leads (27.3%). No significant difference was observed between the groups regarding the lead demographics and the outcome of lead extraction (Table 4).

**Table 4:** Outcome of laser Lead extraction

	Total	Venous occlusion group	Control group
*Outcome per lead*			
Success, n (%)	409 (91.5)	24 (80%)	48 (96%)
Failure, n (%)	38 (8.5)	6 (20%)	2 (4%)
*Outcome per patient*			
Complete Success, n (%)	197 (90.0)	11 (84%)	25 (96.1%)
Partial success, n (%)	17 (7.8)	2 (15.4%)	1 (3.9%)
Failure, n (%)	5 (2.3)	0	0

As seen in Tables 5 and 6, the patients had different anticoagulation regime. In the venous occlusion group, only one patient was under oral anticoagulation (Phenprocoumon) and 3 patients presented with symptomatic (pain and swelling) venous occlusion and in the control group, 14 patients were under Phenprocoumon, 2 patients were under Rivaroxaban, 1 patient was under Apixaban. we compared the two groups using Fisher`s exact test in regard to anticoagulation which led to a significant difference (*p* value = 0.047).

**Table 5:** Characteristics of the venous occlusion group

Patient	Operation	Place of occlusion	Evidence of occlusion	Anticoagulation
1	Bilateral Lead laser extraction	Bilateral subclavian veins, brachiocephalic vein and Superior vena cava	Phlebography	Aspirin 100 mg OD
2	Bilateral Lead laser extraction	Bilateral subclavian veins	Phlebography	No postoperative anticoagulation for 10 days due to bleeding
3	Left-sided lead laser extraction	Subtotal left axillary vein	Duplex Sonography	Aspirin 100 mg OD
4	Left-sided lead laser extraction	Left subclavian vein	Duplex Sonography	None
5	Left-sided lead laser extraction	Left subclavian vein	Phlebography	None
6	Left-sided lead laser extraction	Left subclavian vein	Phlebography	None
7	Left-sided lead laser extraction	Left subclavian and internal jugular veins	Phlebography	None
8	Left-sided lead laser extraction	Left subclavian vein	Phlebography	Phenprocoumon
9	Left-sided lead laser extraction	Left subclavian vein	Duplex Sonography and CT-Scan angiography	None
10	Left-sided lead laser extraction	Left subclavian vein	Phlebography	None
11	Left-sided lead laser extraction	Left subclavian vein	Phlebography	None
12	Left-sided lead laser extraction	Left subclavian vein	Guide wire could not be advanced through the vein	None
13	Left-sided lead laser extraction	Left subclavian vein	Phlebography	Aspirin 100 mg OD

**Table 6:** Characteristics of the control group

Patient	Operation	Evidence of patency	Anticoagulation
1	Left-sided lead laser extraction	Phlebography	Rivaroxaban 15 mg OD
2	Left-sided lead laser extraction	Duplex sonography	Phenprocoumon
3	Left-sided lead laser extraction	Duplex sonography	Phenprocoumon
4	Left-sided lead laser extraction	Phlebography	Aspirin 100 mg OD
5	Right-sided lead laser extraction	Phlebography	Phenprocoumon
6	Left-sided lead laser extraction	Phlebography	Rivaroxaban 20 mg
7	Left-sided lead laser extraction	Implantation of new leads	Phenprocoumon
8	Left-sided lead laser extraction	Phlebography	Aspirin 100 mg OD
9	bilateral lead laser extraction	Phlebography	Phenprocoumon
10	Left-sided lead laser extraction	Phlebography	Apixaban 5 mg BD
11	Left-sided lead laser extraction	Implantation of new leads	Phenprocoumon
12	Right-sided lead laser extraction	Phlebography	Aspirin 100 mg
13	Left-sided lead laser extraction	Duplex sonography	Phenprocoumon
14	Left-sided lead laser extraction	Phlebography	Clopidogrel 75 mg
15	Left-sided lead laser extraction	Phlebography	Phenprocoumon
16	Left-sided lead laser extraction	Phlebography	Phenprocoumon
17	Left-sided lead laser extraction	Phlebography	Phenprocoumon
18	Left-sided lead laser extraction	Implantation of a Hickman line	Aspirin 100 mg OD and Enoxaparin 60 mg
19	Left-sided lead laser extraction	Implantation of new leads	Phenprocoumon
20	Left-sided lead laser extraction	Phlebography	Phenprocoumon
21	Left-sided lead laser extraction	Implantation of Demers line	Phenprocoumon
22	Right-sided lead laser extraction	Implantation of new leads	Aspirin 100 mg OD
23	Left-sided lead laser extraction	Phlebography	None
24	Left-sided lead laser extraction	Implantation of new leads	Phenprocoumon
25	Left-sided lead laser extraction	Implantation of new leads	None
26	Left-sided lead laser extraction	Implantation of new leads	None

## Discussion

By expanding the indications for CIED implantations there is an increase in associated complications. The introduction of the Laser lead extraction system has solved some of these complications and therefore making lead removal a simpler procedure. Recently, laser lead extraction became the standard of care for lead extraction with good results [12].

Present adhesion and fibrosis in the venous system of long-lying leads can bear new challenges and complications which have not been described before.

The use of lead laser extraction sheath can cause vessel or organ perforation, vessel injury and embolism of tissue fragment. Any of these complications entail further adverse events such as massive bleeding, cardiac or vascular avulsion or tears, arrhythmias, heart failure or even death [13, 14]. Autopsy findings performed on 78 deceased patients with a CIED found tissue cuffs in 71% of atrial leads and 87% of ventricular leads. The degree of fibrosis depends on the dwell time of the hardware and younger patient age [15]. In our patient cohort, severs complications associated with lead extractions requiring emergency surgery were < 1%.

In our study population, we faced difficulties implanting new CIED in patients who underwent previous laser lead extraction due to occlusion of the venous access. In contrast, patients with oral anticoagulation showed less occlusions after laser extraction. Tarakji et al. described the effect of laser lead extraction on the structures surrounding the extracted leads. They described how removed leads contained remnants of vascular, atrial and ventricular tissue. It can be concluded that laser sheaths create abrasive surfaces in the vascular system after laser lead extraction. These surfaces are exposed to blood flow and thrombus formation is more likely as a natural response to vessel injury.

It is likely that many patients had occluded venous systems prior to extraction. The lead extraction may have temporarily opened a channel in the subclavian vein thus causing collapse of chronic collateral systems thus when the laser created channel subsequently occluded, patients manifest acute vein occlusion. The theory has until now no studies to support it.

This limited study aims to open an eye on a possible complication which may affect the lives of many patients, especially patients who are dependent on CIED in their life. Many CIED patients are anticoagulated due to various reasons, this limits the complication of venous occlusion after laser lead extraction to a smaller group of non-anticoagulated patients. More detailed studies with a larger patient volume are necessary to further investigate the vascular damage after laser lead extraction. A lot of patients are asymptomatic after venous thrombosis, which may play a leading role in concealing such a complication. Luckily the administration of anticoagulants may prevent postoperative thrombosis. However, we need more evidence through a randomized study as our study is limited to a very small group of patients.

## Conclusion

Laser lead extraction may cause venous occlusion, which can prevent future lead implantation but randomised trials would be needed.

## Data Availability

The datasets generated and/or analysed during the current study are not publicly available due to native language of the data but are available from the corresponding author on reasonable request and should be translated at first.

## Abbreviations

CIED: Cardiac implantable electronic devices
NYHA: New York Heart Association
ICD: Implantable cardioverter defibrillator
